# Anisotropic Terahertz Emission from Bi_2_Se_3_ Thin Films with Inclined Crystal Planes

**DOI:** 10.1186/s11671-015-1190-y

**Published:** 2015-12-15

**Authors:** Sun Young Hamh, Soon-Hee Park, Jeongwoo Han, Jeong Heum Jeon, Se-Jong Kahng, Sung Kim, Suk-Ho Choi, Namrata Bansal, Seongshik Oh, Joonbum Park, Jun Sung Kim, Jae Myung Kim, Do Young Noh, Jong Seok Lee

**Affiliations:** Department of Physics and Photon Science, School of Physics and Chemistry, Gwangju Institute of Science and Technology, 123 Cheomdangwagi-ro, Buk-gu, Gwangju, 500-712 South Korea; Department of Physics, Korea University, 145 Anam-ro, Seongbuk-gu, Seoul, 136-701 South Korea; Department of Applied Physics, College of Applied Science, Kyung Hee University, Yongin, 446-701 South Korea; Department of Electrical and Computer Engineering, Rutgers, The State University of New Jersey, 94 Brett Road, Piscataway, NJ 08854 USA; Department of Physics and Astronomy, Rutgers, The State University of New Jersey, 136 Frelinghuysen Road, Piscataway, NJ 08854 USA; Department of Physics, Pohang University of Science and Technology, 77 Cheongam-Ro, Nam-Gu, Pohang, Gyeongbuk 790-784 South Korea; Advanced Light Source, Lawrence Berkeley National Laboratory, Berkeley, CA 94720 USA

**Keywords:** Topological insulator, Bi_2_Se_3_, Thin film, Terahertz emission, 78.68.+m, 68.37.-d, 73.22.-f

## Abstract

We investigate the surface states of topological insulator (TI) Bi_2_Se_3_ thin films grown on Si nanocrystals and Al_2_O_3_ substrates by using terahertz (THz) emission spectroscopy. Compared to bulk crystalline Bi_2_Te_2_Se, film TIs exhibit distinct behaviors in the phase and amplitude of emitted THz radiation. In particular, Bi_2_Se_3_ grown on Al_2_O_3_ shows an anisotropic response with a strong modulation of the THz signal in its phase. From x-ray diffraction, we find that the crystal plane of the Bi_2_Se_3_ films is inclined with respect to the plane of the Al_2_O_3_ substrate by about 0.27°. This structural anisotropy affects the dynamics of photocarriers and hence leads to the observed anisotropic response in the THz emission. Such relevance demonstrates that THz emission spectroscopy can be a sensitive tool to investigate the fine details of the surface crystallography and electrostatics of thin film TIs.

## Background

Topological insulators (TIs) behave as a charge-gapped insulator in their interior but hosting a spin-momentum-locked Dirac state at the surface. When the Fermi level crosses over a conduction/valence band, a bulk charge transport can overwhelm the surface contribution, so that thin film TIs have been highlighted as a method to reduce bulk carrier effects due to a large surface to bulk volume ratio [1–6]. During extensive research activities on thin film TIs, several experimental techniques have been employed to characterize thin film properties; for example, atomic force microscopy and transmission electron microscopy could reveal the existence of twin domains with a triangular shape and an inversion symmetry breaking even in the bulk state of film TIs [7–11]. Also, surface-sensitive techniques can be utilized to characterize a Dirac dispersion of the surface state; Fermi surface information can be extracted from Shubnikov-de Haas oscillations, and in particular, the interface state formed at the junction between TI and conventional semiconductors can be studied by using tunneling spectroscopy under magnetic field [12–17].

Terahertz (THz) spectroscopy also can provide useful information about the surface state of the TIs. From conventional THz time-domain spectroscopy, Aguilar et al. could retrieve optical response functions, such as optical conductivity, of the TI surface state, and determine electrodynamic parameters of Dirac fermions [18, 19]. Similar information could be obtained from THz emission spectroscopy [20]. Whereas the THz wave can be emitted from the acceleration of photocarriers generated by an illumination of a pulsed laser onto TIs, a change in THz intensity with a variation of the bulk carrier density could be satisfactorily explained by considering the contribution of Dirac fermions together with bulk charge carriers, which then provided useful information about the mobility of surface carriers.

In this paper, we demonstrate that time-domain THz emission measurement can be a sensitive method to investigate the details of the surface crystallography and electrostatics of thin film TIs. We examine an azimuth-dependent THz radiation emitted from Bi_2_Se_3_ thin films grown on Si nanocrystals (NCs) and Al_2_O_3_ and also from the bulk Bi_2_Te_2_Se. Whereas the bulk crystalline Bi_2_Te_2_Se shows an isotropic behavior of the THz emission, film TIs exhibit contrasting behaviors in the phase and amplitude of emitted THz radiation. In particular, the Bi_2_Se_3_ film grown on the Al_2_O_3_ substrate exhibits a strong modulation of the THz electric field profiles upon the variation of the sample azimuth. We will discuss the details of such emitted THz waves from the surface of Bi_2_Se_3_ films by considering the anisotropic dynamics of the photocarriers under the structural anisotropy in the thin film TI.

## Methods

High-quality Bi_2_Se_3_ films with 30–33-nm thickness were grown on Si NCs [9, 21–24] and Al_2_O_3_ by a molecular beam epitaxy (MBE) system. Si NCs were fabricated by annealing SiO_x_/SiO_2_ multilayers grown on Si wafers by ion beam sputtering, whose detailed processes were described in previous reports [21]. The average size of Si NCs used in this work was estimated to be ~3.1 nm by transmission electron microscopy [22, 24]. As a control, we prepared a single crystalline Bi_2_Te_2_Se sample using the self-flux method with a stoichiometry of chunks (Bi:Te:Se = 2:1.95:1.05) [25, 26].

**Fig. 1:**
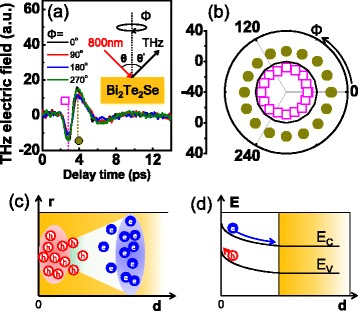
Terahertz (THz) emission results from bulk Bi_2_Te_2_Se and a schematic of THz generation mechanisms. **a** Time profile of the THz electric field emitted from Bi_2_Te_2_Se obtained with a variation of the sample azimuth *Φ* from 0° to 270°. The inset shows a schematic of the THz generation experiment. **b** *Φ*-dependence of maximum (*filled circle*) and minimum (*open square*) amplitudes indicated in (**a)**. **c**, **d** THz generation mechanism by two surge current contributions, i.e., the photo-Dember effect and photocarrier acceleration by surface band bending, respectively. The horizontal axis means the depth *d* from the surface. The vertical axes in (**c**) and (**d**) indicate a lateral dimension in the sample and an electron energy *E*, respectively. *e* (*h*) represents electron (hole). *E*_F_ and *E*_C_ (*E*_V_) lines denote a Fermi energy and conduction band minimum (valence band maximum) of the bulk state

For THz emission spectroscopy, we use 70-fs laser pulses with a center wavelength of 800 nm generated from a Ti:sapphire oscillator at a repetition rate of 80 MHz. The beam is focused onto the sample with a 300-μm spot size at an incidence angle of 45° (inset of Fig. 1a) and a power density of about 0.6 kW/cm^2^, which is below the damage threshold [14]. Because the penetration depth of incident light is about 20 nm [14], THz wave generation takes place near the surface of the sample. Emitted THz radiation is guided by a pair of parabolic mirrors in a specular reflection geometry. The transient electric field of the THz wave is then detected by a photoconductive antenna made of the low-temperature-grown GaAs [27].

Although thin films were grown along the *c*-axis in a hexagonal setting of the crystal, we perform the x-ray diffraction experiment with three degrees of freedom, i.e., *θ*, 2*θ*, and *Φ* motion (Fig. 3a) to characterize the orientation of the crystal plane more accurately. In the geometry of an asymmetric reflection, we tilt the sample using the *θ* motion with respect to the surface normal until we find a maximum intensity of the Bragg peak (006) of Bi_2_Se_3_ with the 2*θ* motion fixed to *θ*_B_ as indicated in Fig. 3a. The same procedure is taken for the substrate diffraction peak. The difference in the tilting angle *θ* between the film and substrate at the same azimuth angle *Φ* corresponds to the inclination angle (Δ*φ*) of the film Bragg plane with respect to the crystal plane of the substrate.

## Results and Discussion

We examine first the THz emission from the bulk crystalline Bi_2_Te_2_Se of which results are displayed in Fig. 1a, b. Time profiles of the THz electric field (Fig. 1a) are obtained by varying the sample azimuth *Φ*, and they commonly show a single-cycle oscillation with a well-defined crest and trough. The THz signals at these points marked by an open square and closed circle are plotted as a function of *Φ* in Fig. 1b. It is clear that the THz amplitude does not change with varying *Φ*, in agreement with the previous report for bulk crystalline Bi_2_Se_3_ [20]. Such an isotropic response of the emitted THz wave indicates that the dominant THz generation mechanism is not an optical rectification but a surge current related to the photo-Dember effect and/or surface band bending. Photocarriers excited by a femtosecond laser form a transient electric dipole moment along the surface-normal direction due to a difference in mobilities of the electron and hole (photo-Dember effect) or an acceleration of charged particles by a surface band bending, which are schematically shown in Fig. 1c, d, respectively. The photo-Dember effect is dominant for narrow-bandgap semiconductors, such as InAs, InSb, and also Bi_2_Te_2_Se studied here, since the remaining energy after absorption will raise the carrier temperature enhancing this effect [28]. Also, it is well known that a charge distribution is inhomogeneous at the surface of TIs resulting from the surface band bending [29]. Therefore, although we could exclude the optical rectification as a mechanism of THz emission in Bi_2_Te_2_Se, it is not clear yet which has a more dominant contribution to the observed THz emission signals between the photo-Dember effect and surface field acceleration.

**Fig. 2:**
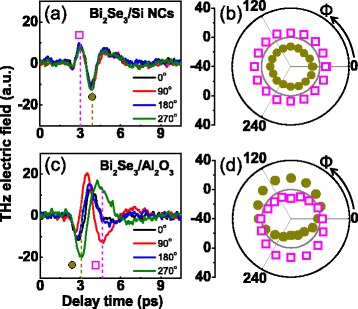
THz emission experiment results from the Bi_2_Se_3_ thin films. **a** Time profile of the THz electric field emitted from the Bi_2_Se_3_ film grown on Si NCs with the azimuth angle *Φ* varying from 0° to 270°. **b** *Φ*-dependence of maximum (*open square*) and minimum (*filled circle*) amplitudes indicated in **a**. **c** Time profile of the electric field emitted from the Bi_2_Se_3_ film grown on Al_2_O_3_. **d** *Φ*-dependence of amplitudes at 3 ps (*filled circle*) and 4.5 ps (*open square*) with a largest amplitude modulation

Figure 2a shows a THz time profile emitted from the thin film Bi_2_Se_3_ grown on Si NCs. Compared to the case of the bulk sample, the amplitude of the THz wave is comparable and the azimuth dependence is similarly isotropic (Fig. 2b). On the other hand, a phase of the emitted THz wave is turned out to be opposite. When the THz wave is generated by photo-Dember effect, its phase is expected to remain the same in a similar kind to the materials where the relative difference in the electron and hole mobilities remains unchanged under the variations of the chemical composition or the carrier concentration. On the other hand, the direction of the built-in electric field or the surface band bending can be developed differently depending on the major carrier type which can change the direction of the photocarrier acceleration and accordingly the phase of emitted THz wave. Therefore, the surface field acceleration rather than the photo-Dember effect would be a more dominant mechanism of THz emission in the Bi_2_Se_3_ thin film and possibly in the Bi_2_Te_2_Se single crystal as well. Accordingly, these two systems can have different situations related to the surface band bending.

Interestingly, the Bi_2_Se_3_ film grown on the Al_2_O_3_ substrate shows more distinctive behaviors in its THz emission response. Differently from the former two cases, the phase of the THz electric field profile is not well defined under the variation of the sample azimuth *Φ* (Fig. 2c) We pick up the amplitudes around 3 ps (filled circle) and 4.5 ps (open square) and display them in Fig. 2d as a function of *Φ*. Although they have a circular shape, the circles themselves are displaced from the center of the coordinates. The off-centering of two circles signifies the existence of a *Φ*-dependent onefold anisotropy affecting THz wave radiation. We can exclude a possible contribution of the optical rectification from the surface state of the 3*m* point group as it should exhibit a sixfold (or threefold) symmetry. Rather than such an intrinsic inversion symmetry breaking at the surface of Bi_2_Se_3_, we pay attention to an additional symmetry breaking possibly induced during the growth process of Bi_2_Se_3_ thin films on the substrate.

**Fig. 3:**
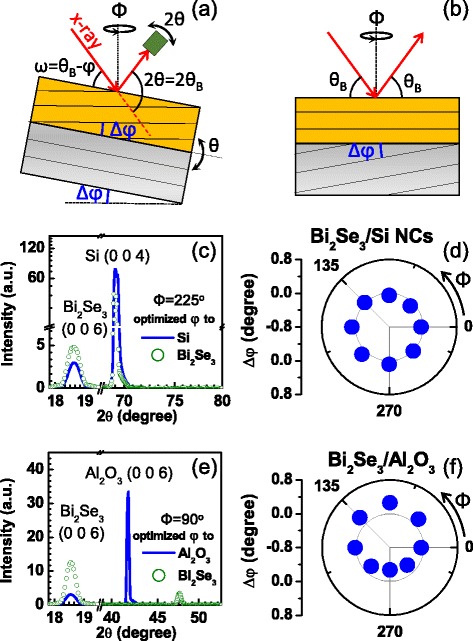
X-ray diffraction experiment for the Bi_2_Se_3_ thin films in an asymmetric reflection geometry. **a**, **b** X-ray diffraction experiment scheme when the Bragg plane of the film is inclined by Δ*φ* with respect to the substrate crystalline plane. **c**, **d** X-ray diffraction experiment results for the Bi_2_Se_3_ film grown on Si nanocrystals (NCs) made on the Si substrate. **c** shows Bragg peaks of Bi_2_Se_3_ (006) and Si (004) obtained through *ω*-2*θ* scans with different *φ* optimized to Bi_2_Se_3_ (006) (*open circle*) and Si (004) (*line*). The inclination Δ*φ* is plotted in **d** as a function of the sample azimuth *Φ*. **e**, **f** X-ray diffraction experiment results for the Bi_2_Se_3_ film grown on the Al_2_O_3_ substrate. Bragg peaks of Bi_2_Se_3_ (006) and Al_2_O_3_ (006) are obtained through *ω*-2*θ* scan with optimized *φ* to Bi_2_Se_3_ (006) (*open circle*) and Al_2_O_3_ (006) (*line*) in **e**. The inclination Δ*φ* is plotted in **f** as a function of the sample azimuth *Φ*

Using the x-ray diffractometer, we examine the relative crystalline directions of the Bi_2_Se_3_ films and the substrate. Figure 3c displays Bragg peaks near 2*θ* = 18.6° corresponding to a (006) peak of Bi_2_Se_3_ grown on Si NCs and near 2*θ* = 69.2° for the (004) peak of a Si substrate beneath the Si NC layer. The sample azimuth *Φ* is set to 225°. It should be noted that the maximum intensity of each peak cannot be obtained simultaneously in a single diffraction geometry. When *φ* is optimized to Si (004), the diffraction peak for Bi_2_Se_3_ (006) (line) has a smaller intensity (by about 20 %) than that of the peak obtained after the optimization to itself (open circle). *φ* is obtained by varying *Φ* for both the film and the substrate, and their difference Δ*φ* is plotted in Fig. 3d. It shows a slightly off-centered circle in the *Φ* variation where the largest deviation from zero value (gray line) appears near *Φ* = 45° and 225° with about Δ*φ* = −0.1° and +0.1°, respectively. This angle corresponds to the degree of inclination of the Bragg plane in the Bi_2_Se_3_ thin film with respect to the substrate Bragg plane.

We employ the same procedures to Bi_2_Se_3_ films grown on an Al_2_O_3_ substrate where the angle *φ* for the Al_2_O_3_ substrate is optimized to its (006) diffraction peak around 2*θ* = 41.7°. At the sample azimuth *Φ* = 90°, the Al_2_O_3_ (006) peak can be optimized to have a maximum intensity such as a line curve in Fig. 3e, but it shifts rightwards with a large reduction of its intensity when *φ* is optimized to the Bi_2_Se_3_ (006) peak. Note that the estimated inclination angle from the azimuth-dependent Δ*φ* shown in Fig. 3f is about 0.27°, and this is about 2.5 times larger than for the film grown on the Si NCs. Typically, Al_2_O_2_ substrates can have a larger vicinal angle than that of a Si wafer. When Bi_2_Se_3_ films are grown on such vicinal substrates, the crystallographic orientation of the films may not follow that of the substrate, and the tilting between the film and the substrate in their *c*-axis orientations can be proportional to the substrate vicinality.

**Fig. 4:**
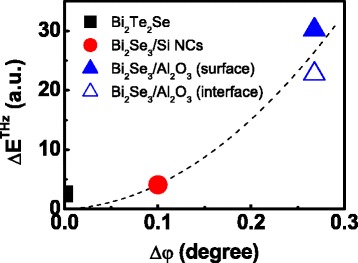
THz amplitude modulation Δ*E* ^THz^ as a function of the Bragg plane inclination angle Δ*φ*. The results are displayed for three samples, i.e., Bi_2_Te_2_Se single crystal and Bi_2_Se_3_ thin films grown on Si NCs and Al_2_O_3_. Note that the result from the interface between the Bi_2_Se_3_ and the Al_2_O_3_ substrate is included. The d*otted line* is merely a guide to eyes

It is intriguing to note that such an inclination of the crystal plane of the film is intimately connected to the aforementioned modulation in the THz electric field. We estimate the modulation amplitude of the THz electric field Δ*E*^THz^ with a variation of *Φ* from the curves given in Figs. 1b and 2b, d. Certainly, Δ*E*^THz^ is negligible for the bulk Bi_2_Te_2_Se and thin film Bi_2_Se_3_ on Si NCs whereas it is relatively large for the film on Al_2_O_3_. This THz-response Δ*E*^THz^ is plotted versus Δ*φ* in Fig. 4 where the Δ*φ* of the bulk Bi_2_Te_2_Se is set to zero. From this plot, we can reasonably expect a possible relationship between these two quantities. To support this hypothesis, it is worthy to note a similar phase relationship between the THz amplitude and Δ*φ* in their *Φ* dependences shown in Figs. 2d and 3f, respectively; they both have a onefold symmetry, and the maximum deviations of each quantity from an isotropic response occur near the same azimuth.

Actually, when the crystal plane is inclined with respect to the sample surface (Fig. 3a), the photocarrier dynamics and consequently the characteristics of the emitted THz waves will be influenced by such a onefold in-planar anisotropy. As a quasi-two-dimensional system, Bi_2_Se_3_ can have an anisotropic behavior along the *c*-axis with respect to an *ab*-plane, such as a dc-conductivity ratio *σ*_ab_/*σ*_c_ of about 3–4 [30]. When the crystalline *ab*-plane is inclined from the sample surface, the surface-normal direction will be defined with contributions from both the crystallographic *c*-axis and the *a*- (or *b*-) axis even though the latter will have a minor contribution. Then the photocarriers generated at the surface, which otherwise are accelerated simply along the surface-normal direction, will experience an anisotropic electrostatic potential or anisotropic transport behavior as they travel inside of the film. Consequently, THz light emitted through a surge current can have the finite anisotropic response on the sample azimuth. Since x-ray diffraction results revealed the relative tilting angle between diffraction planes of the TI film and the substrate, crystallographic planes can be drawn differently as in Fig. 3b where the crystal plane of the substrate is inclined with respect to the sample surface and the TI crystal plane is in parallel with the sample surface. We consider that the configuration in Fig. 3a instead of that in Fig. 3b is more preferable to explain the anisotropic THz emission results since the THz emission in the case of Fig. 3b should appear isotopically as a function of *Φ*. Although the anisotropic THz responses in the variation of *Φ* were discussed in terms of the surge current THz emission process, it should be noted that the results would be explained also by the optical rectification process by considering the lowered surface symmetry.

## Conclusions

We demonstrate that the terahertz (THz) emission spectroscopy can be a sensitive tool to investigate the fine details of the electrostatics and surface crystallography of topological insulator thin films. For the Bi_2_Se_3_ thin film grown on Si nanocrystals, the emitted THz electric field has an opposite phase to that from the bulk Bi_2_Te_2_Se crystal, and this can be attributed to different electrostatics related to the surface band bending of two samples. For the Bi_2_Se_3_ thin film grown on Al_2_O_3_, the THz emission occurs with a strong modulation particularly in its phase upon the variation of the sample azimuth. Through x-ray diffraction experiment, we found that the crystal plane of the film is inclined with respect to the substrate crystal plane, and the resultant planar anisotropy is responsible for the onefold symmetry in the anisotropic THz response. As topological insulators in the form of the thin film have been attracting much attention these days to exploit the Dirac fermionic surface state with less contribution of conventional bulk carriers, we expect that phase-sensitive THz emission spectroscopy can have a clear contribution in such researches by elucidating the lateral and longitudinal electrostatics related to the band bending as well as the structural symmetry at the surface and/or interface of the film.

## Abbreviations

THz: Terahertz
TI: Topological insulator
